# Casual Associations and Shape Between Prepuberty Body Mass Index and Early Onset of Puberty: A Mendelian Randomization and Dose–Response Relationship Analysis

**DOI:** 10.3389/fendo.2022.853494

**Published:** 2022-03-14

**Authors:** Jiao Fang, Jingyi Yuan, Dandan Zhang, Wanxu Liu, Puyu Su, Yuhui Wan, Zhihua Zhang, Fangbiao Tao, Ying Sun

**Affiliations:** ^1^ Department of Maternal, Child & Adolescent Health, School of Public Health, Anhui Medical University, Hefei, China; ^2^ Anhui Provincial Key Laboratory of Population Health & Aristogenics, Anhui Medical University, Hefei, China; ^3^ Department of Epidemiology and Biostatistics, Anhui Medical University, Hefei, China

**Keywords:** body mass index, puberty, Mendelian randomization, causal effects, restricted cubic spline

## Abstract

**Background:**

There is an ongoing controversial issue regarding whether onset of puberty is related to childhood BMI.

**Objectives:**

This study aims at investigating the causal association and its shape between prepuberty BMI and early puberty onset.

**Methods:**

Breast development and testicular volume were assessed annually from a population-based prospective cohort of 997 children for consecutive years by professional endocrinologists. Seventeen puberty- and BMI-related SNPs were selected to calculate the polygenic risk score. The two-stage least square method was used to assess and confirm causal effects. A dose–response association between prepuberty BMI and early puberty onset was conducted by using restricted cubic spline Cox regression.

**Results:**

After adjusting for covariates, prepuberty BMI was positively associated with early thelarche among girls (coefficients = 0.18, 95% *CI*: 0.01, 0.29). A non-linear model suggested an inverted U-shaped relationship between prepuberty BMI and risk for early thelarche (*χ^2^* = 276.3, *p* < 0.001). The risk for early thelarche increased rapidly from prepuberty BMI at 15.70 kg/m^2^ (P_25_) to 20.75 kg/m^2^ (P_85_) and gradually decreased afterward. Compared with the P_25_ of prepuberty BMI, the HRs (95% *CI*) for early thelarche were 5.08 (1.15, 8.55), 4.48 (1.02, 7.74), 10.15 (3.93, 17.50), and 8.43 (1.91, 13.71) for percentiles P_25_–P_50_, P_50_–P_75_, P_75_–P_85_, and ≥P_85_ of BMI categories, respectively. In boys, compared with the P_25_ of prepuberty BMI, boys with BMI between P_25_ and P_50_ showed an increased risk of early puberty (HR: 3.94, 95% *CI*: 1.44, 6.80).

**Conclusions:**

Prepuberty BMI may serve the purpose of identifying the girls at higher risk of early thelarche, which could assist in the adaptation of prevention and intervention strategies targeting childhood obesity. The findings emphasize a non-linear correlation between prepuberty BMI and early puberty onset.

## Introduction

Puberty is a milestone of life phase, with growth and development of all psychological and physiological systems, especially sexual maturation occurrence and reproductive capacity (1). From the middle of the 20th century until now, a temporal trend toward earlier puberty has been observed around the world (2, 3), although it is less certain for gonadarche in boys (4–6). This is a common concern, as early timing of puberty has a wide range of serious health complications, including increased risk of cardiometabolic diseases, depression, cancers, and possible obstetrical and gynecological problems (7, 8). During the same period, the prevalence of obesity in childhood and adolescence has increased substantially worldwide and become a major public health problem (9). Imperial London and the World Health Organization (WHO) reported a ten-fold increase in the number of adolescents and children with obesity aged 5 to 19 years (10). In developed countries during the years of recent economic crises (last decade), the rising trend in body mass index (BMI) or obesity in children and teenagers has been observed to plateau in high-income countries (11), and there is even a small amount of evidence suggesting a statistically significant reduction in overweight and obesity in Greek schoolchildren aged 6–16 years in both sexes (12) but which continues to increase in low- and middle-income countries (LMICs), especially in populous nations like China (11).

Some researchers have suggested the long-term trend of early puberty to be partly attributed to the rise in childhood obesity (13–15). Longitudinal epidemiological studies show that high BMI is related to earlier pubertal maturation in girls (16, 17), specifically that girls with obesity have earlier age at menarche and timing of thelarche than girls with normal weight (14, 15, 18, 19), while a few studies have not found this association (20). In boys, there have been fewer studies; some evidence reported a positive correlation between BMI or obesity and pubertal development in boys (5, 21–23), while some studies indicate a negative association or fail to find any associations (24, 25).

Mendelian randomization (MR) is an alternative means of assessing the causal effect of childhood BMI on early puberty, designed as a quasi-experimental study that is less susceptible to confounding effects. However, to date, applications of MR have been limited to linear models for the associations between exposure and outcome. A clear determination of the shape in the casual relation between childhood BMI and early onset of puberty would elucidate the comparative relevance of higher and lower BMI values on risk for early onset of puberty.

Based on a 4-year prospective cohort with annual objective assessment of pubertal development, the present study aims at validating the causal association and its shape between prepuberty BMI and early onset of puberty in both sexes, by using single-sample MR as well as restricted cubic spline Cox regression.

## Methods

### Study Population

As illustrated in **Supplementary Figure A1**, the perspective puberty cohort is a database containing questionnaires and physical examination information on nearly a thousand school-age children from 2 elementary schools, which was established since March 2016 by clustering convenience sampling in Bengbu, Anhui province, China.

All children underwent a questionnaire survey and physical and pubertal development assessment annually. In 2017, buccal cheek swabs were collected from all the children for DNA extraction and genotyping. The final analysis sample of children who had complete and effective data on BMI status, breast Tanner stage, testicular volume, and genetic susceptibility included 997 children (579 girls and 418 boys) aged 9.33 to 12.17 years in the last follow-up (for detailed information, see Sun et al., 2019) (26). The exclusion criteria were as follows: children with organic or chronic diseases that could affect puberty, or those taking oral or inhaled glucocorticoids or human growth hormone, were excluded in this cohort. Ethical approval for this study was obtained from the Institutional Review Board at Anhui Medical University (No. 20180082). Informed consent for all collected data was obtained from parents and schoolteachers, as well as children.

### Measurements

#### Early Onset of Puberty

Annual breast Tanner assessments were classified between 1 (prepubescent state) and 5 (full sexual maturity state) (27, 28) through both observation and palpation in primary school while testicular volume was estimated by using a Prader orchidometer by trained pediatric endocrinologists. Pubertal onset was defined as attaining breast Tanner stage 2 (B2, thelarche), or testicular volume more than 3 ml (TV4). The 25th percentile was adopted as the cutoff point (*P*_25_ age) for early onset of thelarche and testicular development in Chinese children based on data from the “China Puberty Collaboration Study (29, 30),” which was age at B2 < 8.0 y for girls and age at TV4 ml < 9.7 y (TV3 ml < 8.67 y) for boys.

#### Body Mass Index and Classification

In the annual follow-up survey, height and weight were measured by trained and certified medical staff. Body height was examined with light clothing to the nearest 0.1 cm by using a portable stadiometer, and weight with an electronic scale (Tanita TI1618) to the nearest 0.1 kg. BMI is a measurement of height-adjusted measure of weight, calculated as weight (in kilograms) divided by the square of height (in meters) (kg/m^2^). Childhood BMI was derived from all paired height and weight measurements in the age of 6.5 to 12.5 years and age adjusted to 9 years by using a linear regression model. Moreover, it was classified according to percentiles (< P_10_, P_25_–P_50_, P_50_–P_75_, P_75_–P_85_, and ≥ P_85_).

#### Genotyping and Single-Nucleotide Polymorphism Selection

Genomic DNA for all children had been extracted from buccal cheek swabs following a standard protocol. PCR-RFLP and real-time PCR were used to extract DNA and then genotyped in the Sequenom MassARRAY. The mean concordance rates of the genotyping system were 98.8%.

The present study identified 11 and 21 single-nucleotide polymorphisms (SNPs) associated with obesity and early puberty at genome-wide significance in the GWAS datasets from 87,802 women and 35,668 children of European ancestry, respectively (31–33). After excluding five SNPs (rs35327298, rs142058842, and rs4237264 for early puberty; s7550711 and rs13387838 for obesity) with MAFs < 5%, three SNPs (rs5932886 for early puberty; rs1310484 and rs987237 for obesity) had a genotyping rate <10%. Additionally, we have imputed genotype data based on the CHB HapMap data (Phase 2 and Phase 3) and further verified that loci were not in a significant linkage disequilibrium (LD) with each other (r^2^ < 0.3) in the SNP server and Han Chinese (CHB) data. Moreover, 17 puberty-related SNPs and 7 BMI-related SNPs were retained in the final analysis and the polygenic risk score (PRS) was calculated, as shown in the following formula, respectively:


$$\text{Puberty}-\text{related PRS}={\text{SNP}}_{1}+{\text{SNP}}_{2}+{\text{SNP}}_{3}+\ldots \;+{\text{SNP}}_{17},$$

$$\text{BMI}-\text{related PRS}={\text{SNP}}_{1}+{\text{SNP}}_{2}+{\text{SNP}}_{3}+\ldots \;+{\text{SNP}}_{7}$$

Each SNP was recorded as 0, 1, and 2 according to number of effect alleles (e.g., if the effect allele is T, then TT = 2, CT = 1, CC = 0).

### Covariates

For assessment of potential confounding factors, the current analysis included delivery mode (vaginal or caesarean section), gestational age (>37 weeks or ≤37 weeks), birthweight, and infancy feeding mode (included exclusive breastfeeding, mixed feeding, and formula feeding), as well as parental BMI, education, and household monthly income (obtained from the parents’ questionnaire at baseline).

### Statistical Analysis

Statistical calculations were performed with Stata (version 14.0) and R version 3.6.2. We used instrumental variable methods to estimate the causal effect between BMI and early onset of puberty through polygenic risk using the Mendelian randomization design. Instrumental variable regression with two-stage least-square (2SLS) methods was performed using the BMI-related PRS as the instrument (34). MR–Egger is often used in sensitivity analysis, i.e., the causality between obesity and early onset of puberty. The shape of the relationship between prepuberty BMI and early onset of puberty was established by using restricted cubic spline (RCS) Cox regression (35) based on five knots (P_5_, P_25_, P_50_, P_75_, and P_95_) of prepuberty BMI and reference BMI of 15.70 kg/m^2^ (P_25_) and 16.10 kg/m^2^ (P_25_) in girls and boys, respectively, generating hazard ratios (HR) [95% confidence intervals (CI)]. Furthermore, we analyzed the association between prepuberty BMI and early puberty by using categorical BMI (BMI < P_10_; P_25_–P_50_, P_50_–P_75_, P_75_–P_85_, and ≥ P_85_ kg/m^2^) with BMI at P_10_–P_25_ as reference.

Final models were adjusted for age, BMI- and puberty-related PRS, parental BMI, parental education, family income, birthweight, delivery mode, infant feeding, and gestational age. 0.05 was defined as a significance threshold of two-tailed *p* values.

## Results

### Participants’ Characteristics

The average cohort follow-up rate from Wave 2 to Wave 4 was more than 90%. Among the 997 children in this cohort, 58% were female, and the mean age was 8.01 (SD, 0.85) years at baseline. The average BMI genetic score (7 SNPs) was 4.61 ± 1.40, 15.32 ± 2.47 for puberty genetic score (17 SNPs), as presented in **Supplementary Table A1**.

Approximately one-fifth of children were classified as overweight and obese at each wave, respectively. **Table 1** shows adiposity and pubertal development in boys and girls during the 4-year follow-up. At wave 1 to wave 4, approximately 9.8%, 14.5%, 22.7%, and 24.5% of girls presented early onset of thelarche, and 0.7%, 1.0%, 6.3%, and 9.9% of boys had early onset of testicular development, respectively.

**Table 1:** Adiposity and pubertal development changes during the 4-year follow-up among all children.

Characteristics	Wave Ⅰ (2016)		Wave Ⅱ (2017)		Wave Ⅲ (2018)		Wave Ⅳ (2019)	
	n	Distribution	n	Distribution	n	Distribution	n	Distribution
**Age, years**	997	8.01 ± 0.85	997	8.97 ± 0.85	997	10.01 ± 0.81	997	11.01 ± 0.82
**Adiposity measurements**								
Overweight	997	220 (22.1)	997	191 (19.2)	997	178 (17.9)	997	200 (20.1)
Obesity	997	267 (26.8)	997	270 (27.1)	997	223 (22.4)	997	225 (22.6)
BMI (kg/m^2^)	997	17.96 ± 2.96	997	18.60 ± 3.26	997	18.81 ± 3.57	997	19.78 ± 3.89
**Pubertal status (boys)**								
Early puberty	418	3 (0.7)	418	4 (1.0)	414	27 (6.5)	405	30 (7.2)
Tanner stage 1 (<3 ml)	418	415 (99.3)	418	405 (96.9)	414	269 (65.0)	405	189 (46.7)
Tanner stage 2 (4–8 ml)	418	3 (0.7)	418	13 (3.1)	414	127 (30.7)	405	165 (40.7)
Tanner stage 3 (9–12 ml)	418	0 (0.0)	418	0 (0.0)	414	18 (4.3)	405	34 (8.4)
Tanner stage 4 (15–20 ml)	418	0 (0.0)	418	0 (0.0)	414	0 (0.0)	405	14 (3.5)
Tanner stage 5 (>20 ml)	418	0 (0.0)	418	0 (0.0)	414	0 (0.0)	405	3 (0.7)
**Pubertal status (girls)**								
Early puberty	579	48 (8.3)	579	76 (13.1)	578	76 (13.1)	572	76 (13.1)
Tanner stage 1 (B1)	579	364 (62.9)	579	206 (35.6)	578	131 (22.7)	572	41 (7.2)
Tanner stage 2 (B2)	579	203 (35.1)	579	254 (43.9)	578	220 (38.1)	572	119 (20.8)
Tanner stage 3 (B3)	579	12 (2.1)	579	99 (17.1)	578	141 (24.4)	572	136 (23.8)
Tanner stage 4 (B4)	579	0 (0.0)	579	20 (3.5)	578	75 (13.0)	572	145 (25.3)
Tanner stage 5 (B5)	579	0 (0.0)	579	0 (0.0)	578	11 (1.9)	572	131 (22.9)

### Casual Association Between Prepuberty BMI and Early Onset of Puberty

The coefficients of the bidirectional MR analysis are presented in **Table 2**. The BMI-PRS created from 7 SNPs showed a positive association with prepuberty BMI (coefficient = 0.17, 95% CI: 0.02, 0.33; *p* = 0.031). The BMI-PRS served as a strong instrument for adiposity, with F statistics of 2.424. The results of 2SLS analysis revealed that prepuberty BMI was associated with early thelarche onset in girls (coefficient = 0.18, 95% CI: 0.01, 0.29; *p* = 0.005). In MR sensitivity analyses, the IVW, Egger, and Median methods provided results similar to those of the 2SLS analysis of early puberty based on TV4 assessment (**Supplementary Table A2**). No causal association was observed between prepuberty BMI and early onset of testicular development in boys (coefficient = 0.07, 95% CI: -0.08, 1.92; *p* = 0.113). Furthermore, we also performed an MR sensitivity analysis based on early puberty in boys assessed by TV3, and again no significant association was observed (coefficient = 0.08, 95% CI: -0.07, 1.88; *p* = 0.365) (**Supplementary Table A3**).

**Table 2:** Summary of coefficients used for bidirectional Mendelian randomization analysis.

Instrumental variables	Genetic score with intermediate trait			Genetic score with outcomes			Two-stage IV analysis (early puberty or Z-BMI)	
	Coefficient (95% CI)	*p*-value	*F*-value	Coefficient (95% CI)	*p*-value	*F*-value	Coefficient (95% CI)	*p*-value
BMI genetic score (7 SNPs)								
Boys	0.14 (-0.12, 0.40)	0.305	0.649	0.01 (-0.01, 0.03)	0.112	3.167	0.07 (-0.08, 1.92)	0.113
Girls	**0.17 (0.02, 0.33)**	**0.031**	**2.424**	**0.03 (0.01, 0.05)**	**0.004**	8.245	**0.18 (0.01, 0.29)**	**0.005**

Adjusted for age, puberty-related PRS, birthweight, delivery mode, infant feeding, family income, parental BMI, and education.

The values in bold indicate statistical significance under the model.

### Non-Linear Relationship Between Prepuberty BMI and Early Puberty

**Figure 1:**
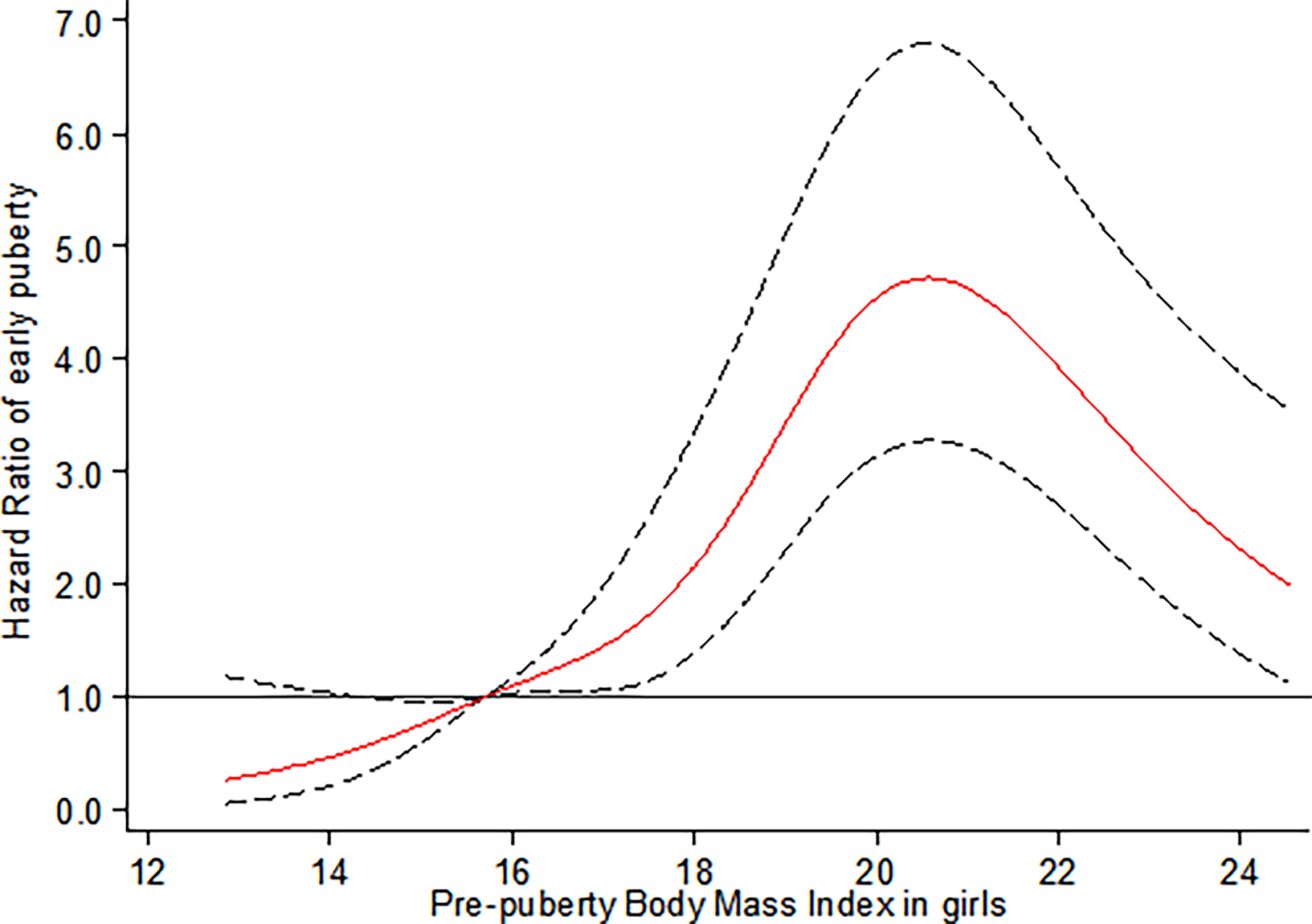
Restricted cubic spline for the association between prepuberty BMI and the HR for early breast development among girls. The curves are based on restricted cubic spline Cox regression with five knots of BMI and a reference BMI of 15.70 kg/m^2^ (P_25_). Individuals with BMI below the 1st or above the 99th percentiles were excluded. Analyses were adjusted for age, BMI- and puberty-related PRS, birthweight, delivery mode, infant feeding, family income, parental BMI, and education.

As illustrated in **Figure 1**, the restricted cubic spline shows that compared to reference groups (prepuberty BMI = 15.70 kg/m^2^, P_25_), girls with elevated percentile of prepuberty BMI before the peak risk point (prepuberty BMI = 20.75 kg/m^2^) are at increased risk for earlier onset of thelarche. After that, the risk for earlier onset of thelarche decreases with a continued increase in prepuberty BMI (*p*_non-linear_ < 0.001).

**Figure 2:**
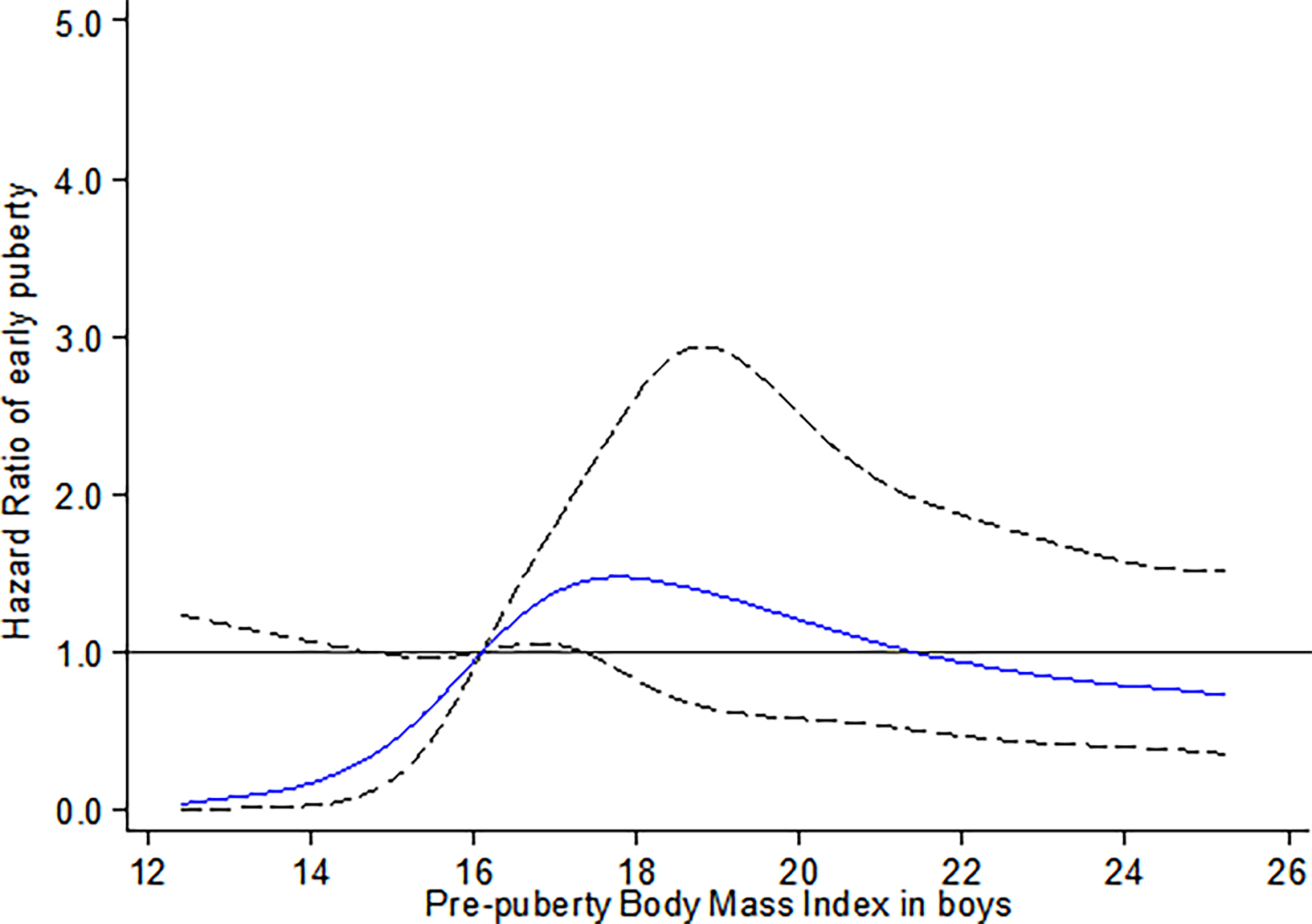
Restricted cubic spline for the association between prepuberty BMI and the HR for early testicular development among boys. The curves are based on restricted cubic spline Cox regression with five knots of BMI and a reference BMI of 16.10 kg/m^2^ (P_25_). Individuals with BMI below the 1st or above the 99th percentiles were excluded. Analyses were adjusted for age, BMI- and puberty-related PRS, birthweight, delivery mode, infant feeding, family income, parental BMI, and education.

For boys, a higher risk of earlier onset of testicular development with prepuberty BMI was observed in the range of 16.10 kg/m^2^ (P_25_) to 17.35 kg/m^2^ (P_40_), compared to the reference group (prepuberty BMI = 16.10 kg/m^2^, *p*_non-linear_ < 0.001) (**Figure 2**).

**Table 3** presents the relationship between categories of prepuberty BMI and risk of early onset of puberty in both sexes. Compared with the reference group (P_10_–P_25_), the HRs (95% CI) for early thelarche were 5.08 (1.15, 8.55), 4.48 (1.02, 7.74), 10.15 (3.93, 17.50), and 8.43 (1.91, 13.71) for BMI categories at P_25_–P_50_, P_50_–P_75_, P_75_–P_85_, and ≥P_85_, respectively, but not in the BMI < P_10_ group. In boys, compared with the reference (P_10_–P_25_), only boys at P_25_–P_50_ of BMI categories showed an increased risk of early onset of testicular development [3.94 (1.44, 6.80)].

**Table 3:** Hazard ratio (95%) of early onset of puberty according to prepuberty BMI in girls and boys.

	Early thelarche in girls (B2)				Early testicular development in boys (TV = 4 ml)			
	No. (%)	Adjusted hazard ratio (95% CI)			No (%)	Adjusted hazard ratio (95% CI)		
		Model 1	Model 2	Model 3		Model 1	Model 2	Model 3
**Prepuberty BMI**								
** <P_10_**	56 (9.7)	**0.79 (0.19, 2.22)**	**0.81 (0.20, 2.30)**	**0.81 (0.24, 2.77)**	39 (9.3)	0.95 (0.21, 2.43)	0.96 (0.21, 2.45)	0.97 (0.24, 1.26)
** P_10_–P_25_**	87 (15.0	Ref.	Ref.	Ref.	65 (15.6)	Ref.	Ref.	Ref.
** P_25_–P_50_**	145 (25.0)	**4.61 (1.05, 7.14)**	**4.95 (1.13, 8.50)**	**5.08 (1.15, 8.55)**	105 (25.1)	1.84 (0.53, 2.87)	1.88 (0.59, 2.94)	**3.94 (1.44, 6.80)**
** P_50_–P_75_**	145 (25.0)	**5.65 (1.31, 9.33)**	**5.02 (1.16, 8.65)**	**4.48 (1.02, 7.74)**	104 (24.9)	1.29 (0.87, 3.89)	1.32 (0.89, 3.97)	2.43 (0.84, 7.00)
** P_75_–P_85_**	58 (10.0)	**9.00 (3.65, 15.70)**	**9.61 (3.64, 15.94)**	**10.15 (3.93, 17.50)**	43 (10.3)	1.59 (0.63, 4.02)	1.59 (0.63, 4.04)	2.52 (0.75, 8.46)
** ≥P_85_**	88 (15.2)	**7.65 (2.07, 12.78)**	**7.72 (1.78, 13.48)**	**8.43 (1.91, 13.71)**	62 (14.8)	0.74 (0.27, 2.04)	0.74 (0.27, 2.03)	1.09 (0.28, 4.21)
*** p* value for trend**		<0.001	<0.001	<0.001		0.406	0.245	<0.001

BMI, body mass index; B, breast; TV, testicular volume.

Model 1 adjusted for age and BMI-related PRS.

Model 2 adjusted for age, BMI- and puberty-related PRS.

Model 3 adjusted for age, BMI- and puberty-related PRS, birthweight, delivery mode, infant feeding, family income, parental BMI, and education.

The values in bold indicate statistical significance under the model.

## Discussion

To our knowledge, this study is the first prospective longitudinal cohort study simultaneously evaluating the causal relationship and its shape between prepuberty BMI with early puberty in both genders. By using Mendelian randomization analysis, we provided robust evidence to support the causal association between increased prepubertal BMI with early onset of thelarche in girls. However, a similar association was not confirmed in boys. For non-linear relations, an inverted U-shaped curve was observed between prepuberty BMI and earlier onset of thelarche. Specifically, girls with elevated percentile of prepuberty BMI before the peak risk point (20.75 kg/m^2^) are at increased risk for earlier onset of thelarche. After that, the risk for early thelarche decreases with a continued increase in BMI. In contrast, for boys, the risk for early onset of testicular development was observed with the prepuberty BMI in the range of 25th to 40th percentile, equal to 16.10 kg/m^2^ to 17.35 kg/m^2^, respectively.

Previous longitudinal epidemiological studies have shown that high childhood BMI is related to earlier pubertal maturation in girls (16–19), while potential correlations among boys are controversial (20–25). Additionally, although BMI and timing of puberty are known to be closely linked, the causality and its effects between these traits in boys and girls remain poorly studied (36).

Our finding of MR analysis in girls is consistent with results from the Taiwan Children Health Study (TCHS) (16), supporting the causal effects of higher adiposity accumulation during childhood on earlier onset of puberty. However, TCHS is limited by using a self-reported questionnaire to define breast Tanner stages. Their measurements of pubertal development starting at ages 11 and 12 lead to underestimation of the true age at pubertal onset in girls. In comparison, our study was based on a 4-year cohort with objective annual breast development assessment since childhood (around 6–8 years) and no more than 2% of girls enrolled at baseline initiated puberty, helping to observe and capture the process of pubertal onset.

The results of the present MR analysis indicated no casual association between prepuberty BMI and early pubertal onset in boys, which is inconsistent with two previous MR studies in boys. The Copenhagen Puberty Study (2006–2014) using a mixed longitudinal cohort (n = 93) and cross-sectional study (n = 637) of 730 healthy Danish boys determined the possible causal link between higher BMI and earlier timing of voice break in boys (37). A longitudinal analysis from the Taiwan Children Health Study (TCHS) also revealed that prepuberty BMI (overweight/obesity) predicted early onset of self-reported pubertal development among male adolescents (17). The heterogeneities could be largely explained by the differences in male pubertal assessment. Although voice break, as a measure of puberty, is frequently used in epidemiological studies, it represents a late pubertal milestone and the validity of self-reported voice break remains a question. As male pubertal onset is manifested by the gradual enlargement of genital and testicular size, direct measurement of testicular volume by palpation is likewise preferable to recalled age at voice break, providing a more accurate estimate of age at attaining the milestones of puberty for boys.

The current study, herein, further extended the non-linear dose–response relationship between prepuberty BMI and onset of puberty by using the restricted cubic spline (RCS) Cox regression model of five knots and indicated a significant non-linear dose–response association of prepubertal BMI with early onset of puberty in both genders. Our finding of an inverted U-shaped correlation with an inflection point in the risk function at 20.75 kg/m^2^ (equal to BMI threshold for obesity at 9 years of age) in girls further complements existing evidence of non-linear associations between prepubertal BMI with early onset of puberty. This is in line with conclusions from the Danish National Birth Cohort (DNBC) and sibling-matched study (38). The DNBC study indicated that childhood overweight (between 85th and 95th percentiles of BMI) and obesity (≥ 95th percentile of BMI ) were associated with earlier puberty timing (self-reported pubertal milestones) in both sexes in a dose-dependent manner by using restricted cubic splines with three knots. A confirmatory analysis of the association was conducted in a sibling-matched study of 1,700 brothers of DNBC, and it reported that a higher BMI was associated with earlier age at attaining most milestones of puberty among girls, but only a tendency toward earlier timing of puberty was observed in boys. Despite the small sample size of the present study, our results highlight the association between objectively assessed BMI and early breast development, which is more convincing than parental report data in the DNBC study.

Our findings in boys supported the results of Bygdell et al. (39) and a DNBC sibling-matched study (38), suggesting that the risk for early testicular development increased with an increasing prepuberty BMI within the range of 16.10 to 17.35 kg/m^2^ (equivalent to the range of normal weight). Bygdell et al. (39) demonstrated that prepubertal BMI associated with early timing of puberty (indicated by age at PHV) in normal-weight but not overweight boys. However, the piecewise linear regression model used in their study cannot observe the shape of the non-linear correlation on both sides of the threshold.

### Potential Mechanisms

The possible mechanisms and relevance of our present findings in terms of sex divergence of early pubertal timing by prepubertal BMI merit further investigation. Understanding of the neurobiological basis of puberty in general, especially the underlying mechanisms for its metabolic regulation in particular, has substantially expanded in recent years. Sanchez Garrido et al. (40) evaluated sexually dimorphic responses in a metabolic programming of puberty to nutritional challenges in rats of both gender. Their study found that male puberty is more sensitive to postnatal nutritional stress (overfeeding) and female puberty is more vulnerable to peripubertal nutritional stress (high-fat feeding) (41). On the other hand, overfeeding before and after puberty leads to an increase in hypothalamic Kiss1 expression and advances the onset of puberty in female rats, whereas sustained excess energy and obesity are associated with inhibition of Kiss1 expression and lower expression levels of key limiting factors of testicular steroidogenesis in male rodents (42).

Human studies further note that excessive adiposity, in the absence of substantial sex steroid surge, may partly suppress hypothalamic–pituitary–gonadal function in girls with earlier puberty, although it is known to accelerate pubertal onset, which indirectly elucidated the decreased risk of early pubertal onset in obese girls than overweight ones, but the mechanism is unclear in boys. Future population-based studies to better characterize the association between body fat and onset of puberty in boys are needed and will require focus on the neuroendocrine basis of the physiological control of puberty and its deviations, as well as epigenetic regulation and metabolic cues, to better understand the mechanisms that trigger pubertal initiation and progression in both sexes.

### Strengths and Limitations

Although the present study is based on a rigorous longitudinal design with repeated objective assessment of puberty, there are some limitations that should be acknowledged. First, this study was conducted among Chinese children; differences between race/ethnicities should be considered for generalizing our findings. Secondly, our analysis used BMI as the primary indicator to measure child obesity, which may not fully reflect body fat level as its calculation only considers height and weight. Further studies are recommended using skinfold thickness or other measures such as dual-energy X-ray absorptiometry to examine the association between body fat and timing of puberty. Third, we only considered the prepubertal BMI of children, which can explain the effects of prepubertal weight status on early puberty; it is necessary to clarify the influence of weight changes during follow-up on puberty in future analysis. In addition, due to the small size of sample, the number of our candidate SNPs representing exposure and outcome phenotype were relatively small compared with large GWAS, which may affect the stable and reasonable estimates of the MR model. Therefore, we also did a further sensitivity analysis using MR–Egger. Given the difficulty of conducting population surveys, another limitation of this study would be that there has been no evaluation of the individual growth charts and the children’s growth patterns related to their target heights and their bone age of course combined at least with basal LH < 08:00 h. Furthermore, although there seems to be no evidence of a secular trend for gonadarche in boys, such a trend seems to be evident for pubarche, and future studies should consider the effect of obesity on adrenarche, especially in boys (4). Some evidence indicated that the physical changes of puberty require a concerted effort from many organs and that these changes are independent of each other, although adrenal maturation often coincides with HPG axis maturation; it is important to note that pubarche itself is not the best indicator of pubertal development. Finally, nearly half of the boys in the cohort had not reached puberty yet at the last follow-up, which may have reduced the power of studies.

## Conclusion

The current study provided robust evidence on the casual and inverted U-shaped relationship between prepubertal BMI with early thelarche in girls. For boys, although no similar causal association was observed, our finding identified a non-linear relationship between BMI and early onset of testicular development in normal-weight boys. Further studies are warranted to elucidate the mechanisms behind the observed associations, which might aid future interventional studies targeting the prevention of childhood obesity and precocious puberty.

## Data Availability Statement

The original contributions presented in the study are included in the article/**Supplementary Material**, further inquiries can be directed to the corresponding author.

## Ethics Statement

We secured approval from the Institutional Review Boards at Anhui Medical University (No. 20180082). Written informed consent to participate in this study was provided by the participants’ legal guardian/next of kin.

## Author Contributions

YS, PS, YW, and ZZ conceptualized and designed the study, drafted the initial manuscript, and reviewed and revised the manuscript. JF, JY, DZ, and WL designed the data collection instruments, collected the data, carried out the initial analyses, and reviewed and revised the manuscript. FT conceptualized and designed the study, coordinated and supervised the data collection, and critically reviewed the manuscript for important intellectual content. All authors approved the final manuscript as submitted and agree to be accountable for all aspects of the work. All authors contributed to the article and approved the submitted version.

## Funding

This work was funded from the National Natural Science Foundation of China (grant number 82173537), National Natural Science Foundation of China (grant number 81872638), Scientific Promotion Project of Anhui Medical University (grant number 2021xkjT012), and Natural Science Foundation of Anhui Province for Distinguished Young Scholars (grant number 1908085J26).

## Conflict of Interest

The authors declare that the research was conducted in the absence of any commercial or financial relationships that could be construed as a potential conflict of interest.

## Publisher’s Note

All claims expressed in this article are solely those of the authors and do not necessarily represent those of their affiliated organizations, or those of the publisher, the editors and the reviewers. Any product that may be evaluated in this article, or claim that may be made by its manufacturer, is not guaranteed or endorsed by the publisher.
