# Supplementary material for: Casual Associations and Shape Between Prepuberty Body Mass Index and Early Onset of Puberty: A Mendelian Randomization and Dose–Response Relationship Analysis
Source: Front Endocrinol (Lausanne). 2022 Mar 14;13:853494. doi: 10.3389/fendo.2022.853494 (PMC8964141; doi:10.3389/fendo.2022.853494)
Supplement: Supplementary file 1 [file DataSheet_1.doc]

**Table A1** Characteristics of school-aged children in this study

| **Characteristics** |  | n | Distribution |
| --- | --- | --- | --- |
| **Gender, boys** |  | 997 | 418 (41.9) |
| **Prepuberty age, years** |  | 997 | 9.03±0.98 |
| **Prepuberty BMI, kg/m^2^** |  | 997 | 18.37±3.30 |
| **Feeding mode** |  |  |  |
| Exclusive breastfeeding |  | 949 | 438 (46.2) |
| Mixed feeding |  | 949 | 352 (37.1) |
| Formula milk |  | 949 | 159 (16.7) |
| **Birthweight, kg** |  | 940 | 3.42±0.47 |
| **Delivery mode, caesarean section** |  | 947 | 459 (48.5) |
| **Gestational age, <37 weeks** |  | 947 | 41 (4.3) |
| **Parental education** |  |  |  |
| Junior school or below |  | 918 | 160 (17.5) |
| High school |  | 918 | 340 (37.0) |
| Vocational college |  | 918 | 248 (27.0) |
| Bachelor or postgraduate |  | 918 | 170 (18.6) |
| **Family income** |  |  |  |
| <5000 |  | 997 | 164 (16.4) |
| 5000-10000 |  | 997 | 548 (55.0) |
| ≥10000 |  | 997 | 285 (28.6) |
| **BMI genetic score (7 SNPs)** |  | 997 | 4.48±1.37 |
| **Puberty genetic score (17 SNPs)** |  | 997 | 15.32±2.47 |

**Table A2** Estimates of causal effect of prepuberty BMI on early onset of puberty at by different Mendelian Randomization sensitivity analysis.

| **Analysis Method** |  | **Girls** | | | |  | **Boys** | | | |
| --- | --- | --- | --- | --- | --- | --- | --- | --- | --- | --- |
|  |  | Coefficient | Standard Error | OR (95%*CI*)  for early puberty | *p*-value |  | Coefficient | Standard Error | OR (95%*CI*)  for early puberty | *p*-value |
| **Inverse-variance weighted** |  |  |  |  |  |  |  |  |  |  |
| **Fixed-effect** |  | 0.09 | 0.02 | 1.09 (0.85, 1.12) | 0.210 |  | 0.12 | 0.03 | 0.89 (0.56, 1.39) | 0.603 |
| **Random-effects** |  | 0.09 | 0.02 | 1.09 (0.85, 1.12) | 0.210 |  | 0.12 | 0.03 | 0.89 (0.56, 1.39) | 0.603 |
| **Egger regression, intercept** |  |  |  |  |  |  |  |  |  |  |
| **Fixed-effect** |  | 0.01 | 0.01 | 1.01 (0.98, 1.02) | 0.320 |  | 0.01 | 0.01 | 1.01 (0.98, 1.02) | 0.390 |
| **Random-effects** |  | 0.01 | 0.01 | 1.01 (0.98, 1.02) | 0.320 |  | 0.01 | 0.01 | 1.01 (0.98, 1.02) | 0.387 |
| **Egger regression, slope** |  |  |  |  |  |  |  |  |  |  |
| **Fixed-effect** |  | 0.03 | 0.08 | 1.08 (0.88, 1.21) | 0.60 |  | 0.00 | 0.09 | 1.10 (0.88, 1.27) | 0.98 |
| **Random-effects** |  | 0.03 | 0.08 | 1.08 (0.88, 1.21) | 0.60 |  | 0.00 | 0.10 | 1.11 (0.82, 1.22) | 0.98 |
| **Simple median** |  | 0.06 | 0.07 | 1.08 (0.92, 1.23) | 0.41 |  | 0.14 | 0.09 | 1.10 (0.96, 1.38) | 0.12 |
| **Weighted mesian** |  | 0.04 | 0.06 | 1.06 (0.94, 1.17) | 0.43 |  | 0.05 | 0.08 | 1.08 (0.91, 1.23) | 0.49 |

**Table A3** Summary of coefficients used for bidirectional Mendelian randomization analysis.

| **Instrumental variables** |  | **Genetic score with intermediate trait** | | | **Genetic score with outcomes** | | | **Two-stage IV analysis**  **(Early puberty ^#^ or *Z*-BMI)** | |
| --- | --- | --- | --- | --- | --- | --- | --- | --- | --- |
|  |  | Coefficient (95%CI) | *p*-value | *F*-value | Coefficient (95%CI) | *p*-value | *F*-value | Coefficient (95%CI) | *p*-value |
| BMI genetic score (7 SNPs) |  |  |  |  |  |  |  |  |  |
| boys |  | 0.13 (-0.03, 0.35) | 0.388 | 0.746 | 0.01 (-0.01, 0.02) | 0.364 | 0.827 | 0.08 (-0.07, 1.88) | 0.365 |
| girls |  | **0.17 (0.02, 0.33)** | **0.031** | **2.424** | **0.03 (0.01, 0.05)** | **0.004** | 8.245 | **0.18 (0.01, 0.29)** | **0.005** |
| Adjusted for age, puberty-related PRS, birthweight, delivery mode, infant feeding, family income, parental BMI and education.  ^#^ Defined as testicular volume >=3ml | | | | | | | | | |

**
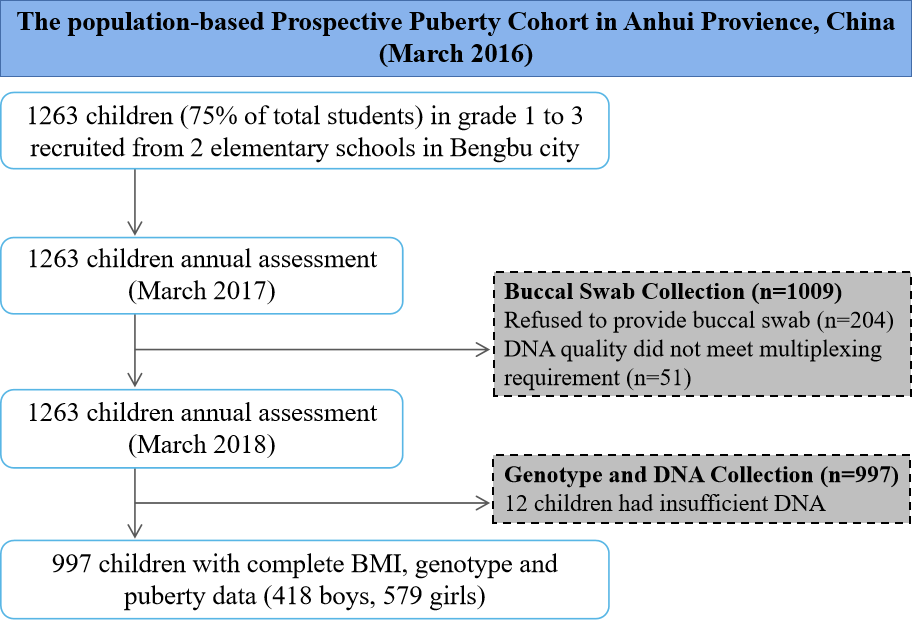
**

**Figure A1**


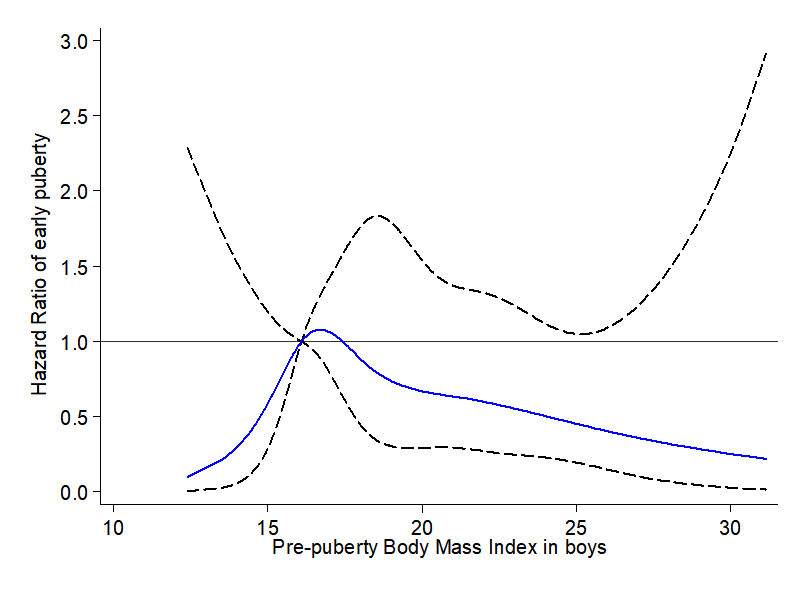


**Figure A2** Restricted cubic spline for the association between prepuberty BMI and the HR for early testicular development among boys defined as testicular volume >=3ml.

Note: The curves are based on restricted cubic spline Cox regression with five knots of BMI and a reference BMI of 16.10 kg/m^2^ (P_25_). Individuals with BMI below the 1st or above the 99th percentiles were excluded. Analyses were adjusted for age, BMI- and puberty-related PRS, birthweight, delivery mode, infant feeding, family income, parental BMI and education.
